# Interpretable automated cobb angle measurement in adolescent idiopathic scoliosis using a lightweight keypoint-to-geometry network: cross-center external validation

**DOI:** 10.3389/fmed.2026.1798211

**Published:** 2026-04-14

**Authors:** Zhiyuan Yang, Jing Qiu, Daozhi Qian, Dongren Liu, Nan Zhang, Ruiyang Yao, Lewei Zhang, Bentao Jiang, Qinghua Meng, Chunyu Bao

**Affiliations:** 1 Tianjin University of Sport, Tianjin, China; 2 Shandong Provincial Maternal and Child Health Care Hospital Affiliated to Qingdao University, Jinan, Shandong, China; 3 Jinan Third People's Hospital, Jinan, Shandong, China; 4 Shandong Sports Rehabilitation Research Center, Jinan, Shandong, China; 5 China Institute of Sport Science, Beijing, China; 6 China Institute of Sports Medicine, Beijing, China

**Keywords:** adolescent idiopathic scoliosis, Cobb angle, deep learning, keypoint detection, YOLO13

## Abstract

Adolescent idiopathic scoliosis (AIS) is routinely quantified on standing coronal radiographs using the Cobb angle; however, manual measurement is time-consuming and subject to intra-observer variability. We propose SpineNET, a lightweight, interpretable keypoint-based framework for automated scoliosis assessment on standing whole-spine posteroanterior (PA) radiographs. SpineNET is built on the YOLOv13 paradigm and enhances cross-scale structural modeling by replacing DS-C3k2 modules with Star-Blocks throughout the backbone and neck, while a lightweight shared-convolution detection head reduces redundancy across feature pyramid levels. The model is trained using only vertebral and pelvic bounding boxes and anatomical keypoints, without any Cobb angle supervision. During inference, Cobb angles as well as shoulder and pelvic coronal tilt angles are computed via a transparent keypoints-to-geometry pipeline with clinically auditable visual overlays. In a cross-center external validation (1,200 radiographs for training/validation and 150 independent external test radiographs), SpineNET achieved 94.2% mAP@50 and 85.7% mAP@50–95 with 1.7M parameters and 4.3B FLOPs. Compared with the consensus of three senior spine surgeons, SpineNET yielded a Cobb angle mean absolute error (MAE) of 1.44° and intraclass correlation coefficient (ICC) of 0.970, with minimal bias (0.03°) and narrow 95% limits of agreement. These results support SpineNET as an efficient and clinically interpretable measurement-assisted tool for high-throughput AIS screening and follow-up in resource-constrained settings.

## 1 Introduction

Adolescent idiopathic scoliosis (AIS) is one of the most common spinal deformities in children and adolescents, with an estimated prevalence of approximately 2%–3% (1). Clinically, scoliosis is typically defined on standing posteroanterior (PA) coronal radiographs by a Cobb angle greater than 10°, and the Cobb angle remains the reference standard for quantifying curve magnitude (1, 2). Beyond severity stratification, Cobb angle measurements are central to clinical decision-making, informing the choice among observation, bracing, and operative management (1, 3). However, conventional Cobb angle measurement requires manual selection of end vertebrae and construction of endplate lines, making the workflow time-consuming and susceptible to subjective variability (4). Reported measurement variability ranges from 2° to 11°, and differences of up to 5° may occur even when the same end vertebrae are selected (5). Consequently, developing robust, interpretable, and deployable automated measurement and decision-support tools has become an important strategy to improve screening efficiency and measurement consistency in AIS (5).

Importantly, radiographic evaluation of AIS extends beyond Cobb angles. Coronal-plane features—such as shoulder height asymmetry, pelvic obliquity, and global coronal balance—are closely related to external appearance, compensatory mechanisms, and treatment planning (6). In particular, pelvic obliquity has been reported to influence radiographic shoulder imbalance and participate in coronal compensatory patterns (7).

Against this background, deep learning has accelerated the development of automated Cobb angle estimation. Existing approaches can be broadly categorized into three paradigms: (1) direct angle regression, (2) spine segmentation followed by geometric fitting, and (3) vertebral landmark/keypoint detection followed by geometric computation (8–11). Direct regression pipelines are computationally simple, but they typically fail to provide auditable evidence for end-vertebra selection and endplate construction, limiting clinical interpretability. Segmentation-based methods, in contrast, rely heavily on mask quality; however, robust segmentation on standing coronal radiographs remains challenging due to low soft-tissue contrast, overlapping anatomical structures, and variable imaging conditions. Relatively, landmark- or corner-based approaches can output clinically reviewable evidence (e.g., end vertebrae and measurement line segments), which better aligns with routine radiographic verification. Nevertheless, these methods are more sensitive to noise and can suffer from a cascade effect—small keypoint deviations may lead to incorrect end-vertebra identification, thereby amplifying Cobb angle errors. Therefore, keypoint-based frameworks intended for real-world deployment require stronger structural modeling capability and more robust cross-domain generalization (12).

From a methodological perspective, keypoint localization in coronal spine radiographs entails pronounced long-range structural dependency and cross-scale consistency. Vertebrae are distributed sequentially along the spinal axis, and stable end-vertebra selection—as well as reliable angle estimation—depends on global inter-segment geometry rather than local texture alone. To enhance modeling of such global spinal structure, we adopt the YOLO13 detection paradigm, which is designed for high-order correlation enhancement and end-to-end feature collaboration in complex scenes, aiming to strengthen cross-scale information interaction and improve model robustness across imaging centers and fields of view (17). Meanwhile, clinical screening and rehabilitation settings are highly sensitive to inference efficiency and deployment cost; thus, the model should maintain localization accuracy while minimizing parameter count and computational overhead (13).

Based on the above needs, we propose SpineNET, a lightweight framework for keypoint detection and angle quantification on standing whole-spine posteroanterior (PA) radiographs in children and adolescents. SpineNET is built upon the YOLOv13 framework, and further optimizes the network for spine radiographic feature extraction by (i) uniformly replacing DS-C3k2 modules in both the backbone and neck with Star-Blocks, and (ii) designing a shared-convolution lightweight detection head for multi-scale key anatomical structures. In this way, lightweight design and representational enhancement jointly cover the full pipeline from feature extraction to multi-scale fusion, improving keypoint localization stability under cross-center and cross-field-of-view (cross-FOV) distribution shifts while reducing inference overhead.

During training, SpineNET learns only vertebral bounding boxes and keypoint localization, without using any Cobb angle labels. During inference, Cobb angles, shoulder coronal tilt angles, and pelvic coronal tilt angles are derived via geometric computation based on predicted keypoints. The system further generates clinically interpretable outputs through visual overlays enabling measurement-assisted decision support. To assess clinical reliability, we compare SpineNET-derived Cobb angles with consensus measurements by three senior spine surgeons, and quantify both error magnitude and agreement, thereby establishing an evidence chain from algorithmic performance to clinical usability.

In summary, our main contributions are as follows:

1. **SpineNET architecture**: Within the YOLO13 framework, we uniformly replace DS-C3k2 modules in both the backbone and neck with Star-Blocks, enabling lightweighting and representational enhancement across the entire feature extraction and multi-scale fusion pipeline, and improving robustness and efficiency under cross-center and cross-FOV settings.
2. **Shared-convolution lightweight detection head**: We propose a lightweight detection head for multi-scale key anatomical structures following a lightweight shared-convolution design (LSCD). The head reduces parameters and FLOPs via cross-scale shared convolutions and improves multi-scale prediction stability and generalization by integrating a Scale layer and Group Normalization (GN).
3. **Cobb-label-free geometry-driven angle quantification**: We established a fully transparent keypoint-to-geometry pipeline without using any Cobb angle labels during model training. During inference, SpineNET first outputs high-precision vertebral corner points, spinous-process landmarks, and bilateral posterior superior iliac spine (PSIS) keypoints. The Cobb angles and ancillary coronal tilt angles are then directly calculated from these predicted keypoints via deterministic geometric operations, forming a clinically auditable and interpretable measurement-assisted system.
4. **Cross-center external validation**: We conduct external testing across centers and systematically compare automatically computed Cobb angles with consensus measurements by three senior spine surgeons, using agreement statistics to support high-throughput workflows in screening and rehabilitation clinics.

## 2 Methods

### 2.1 Data sources and annotation scheme

#### 2.1.1 Data acquisition and dataset partition

This study is a retrospective imaging methodology study. Data were collected from Shandong Sports Rehabilitation Hospital and the Institute of Sports Medicine, General Administration of Sport of China, and comprised standing whole-spine posteroanterior (PA) radiographs from children and adolescents aged 6–18 years. The imaging field of view included three categories—whole spine, thoracic spine, and lumbar spine—which were maintained in an approximately balanced distribution in the overall dataset.

The demographic and clinical characteristics of the training/validation set and external test set are summarized in Table 1, including the distribution of sample size, age, gender, Cobb angle range and imaging field of view (FOV) types. The two datasets showed a balanced distribution of key characteristics, which ensured the reliability of the cross-center generalization performance evaluation of the model.

**Table 1:** Demographic and clinical characteristics of the study datasets.

Characteristic	Training/validation set	External test set
Number of radiographs	1,200	150
Age, years, mean ± SD	12.4 ± 3.1	12.7 ± 2.9
Age range, years	6–18	6–18
Gender, *n* (%)		
Female	782 (65.2)	98 (65.3)
Male	418 (34.8)	52 (34.7)
Cobb angle range, °	5–58	5–46
Cobb angle, °, mean ± SD	22.6 ± 11.3	23.1 ± 10.8
Field of view, *n* (%)		
Whole spine	412 (34.3)	52 (34.7)
Thoracic spine	395 (32.9)	49 (32.7)
Lumbar spine	393 (32.8)	49 (32.6)

In total, 1,200 radiographs were included from Shandong Sports Rehabilitation Hospital and 150 radiographs from the Institute of Sports Medicine. All images were de-identified prior to study entry and underwent standardized format conversion and quality control.

To rigorously assess cross-institution generalization under real-world deployment conditions, we adopted a cross-center external testing design: images from Shandong Sports Rehabilitation Hospital were used for model training and internal validation, whereas images from the Institute of Sports Medicine served as a fully independent external test set. Dataset partitioning was performed at the patient level. The external test set was kept sealed throughout model training and hyperparameter tuning and was used only once for final evaluation after the model was fixed.

#### 2.1.2 Annotation protocol

To enable an interpretable keypoints-to-geometry pipeline, we annotated instance-level bounding boxes and anatomical keypoints as supervision. Vertebral levels followed clinical convention (T1–L5; 17 vertebrae). Because thoracic- and lumbar-view radiographs may have a truncated field of view, we labeled all visible vertebrae in each image; vertebral level alignment and inference were completed during geometric computation using pelvic landmarks.

Specifically, we annotated

1. vertebral bounding boxes for each visible vertebra;
2. pelvic bounding boxes over the bilateral posterior superior iliac spine (PSIS) regions;
3. five vertebral keypoints per vertebra (four corner points to approximate endplate orientation and one midline spinous-process point); and
4. bilateral PSIS endpoints as pelvic landmarks for computing the pelvic coronal tilt angle and anchoring the inferior field-of-view boundary.

**Figure 1:**
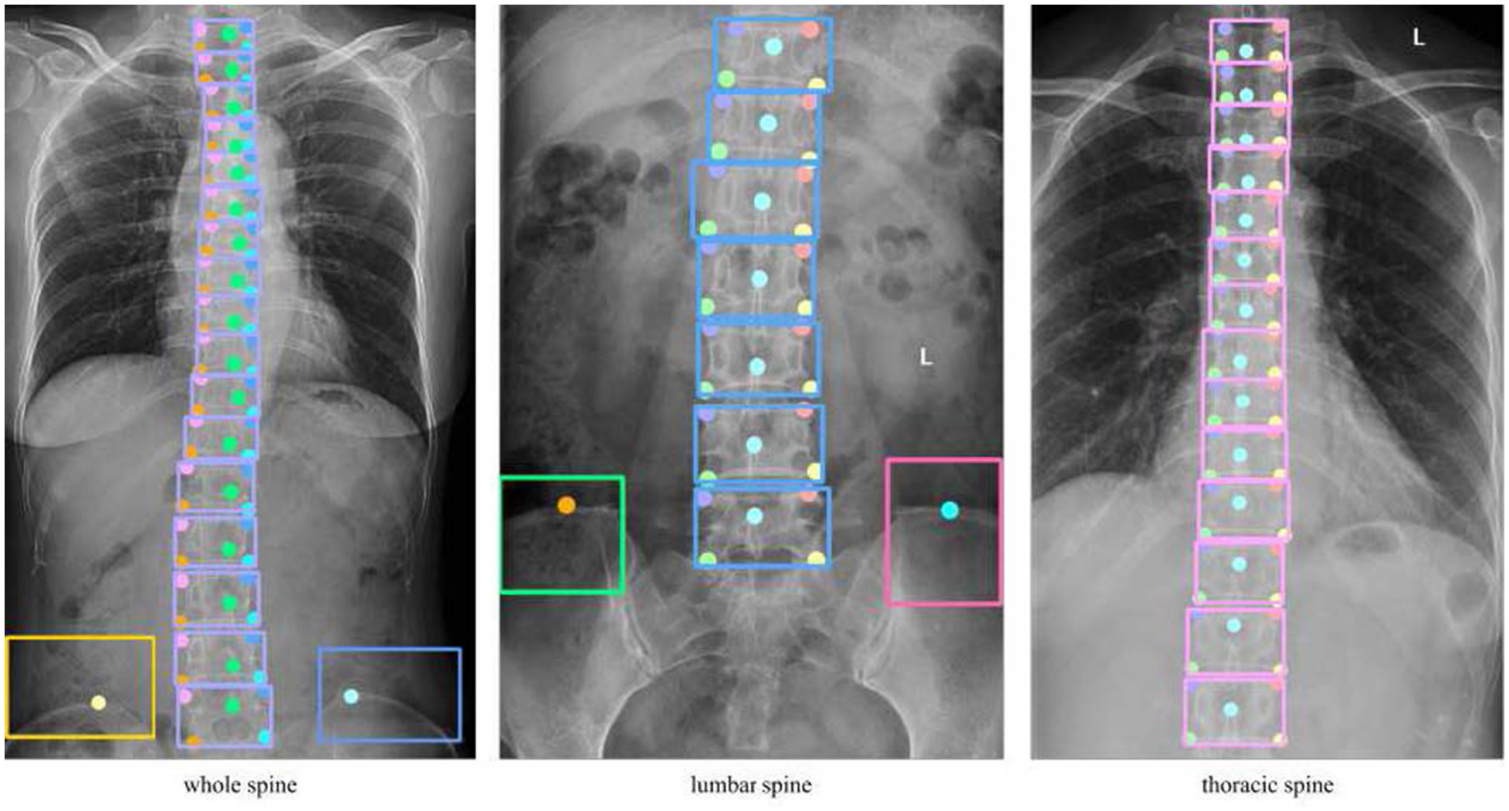
Schematic of keypoint and bounding box annotation. Each vertebra is labeled with a bounding box and 5 keypoints (4 vertebral corners, 1 spinous process midpoint); the pelvic region is labeled with a bounding box enclosing bilateral posterior superior iliac spine (PSIS) endpoints.

All annotations were produced by multiple physicians under a standardized protocol and reviewed by a senior spine surgeon. The annotation format is illustrated in Figure 1.

### 2.2 SpineNET architecture

SpineNET is an end-to-end keypoint detection model that performs vertebral instance detection and anatomical keypoint localization simultaneously. Notably, no Cobb angle or end-vertebra labels are used during training; the model learns only the mapping from the input radiograph to bounding boxes and keypoints. All clinically relevant quantitative measurements are derived during inference via geometric computation, which preserves interpretability and mitigates the auditability limitations associated with direct angle regression.

**Figure 2:**
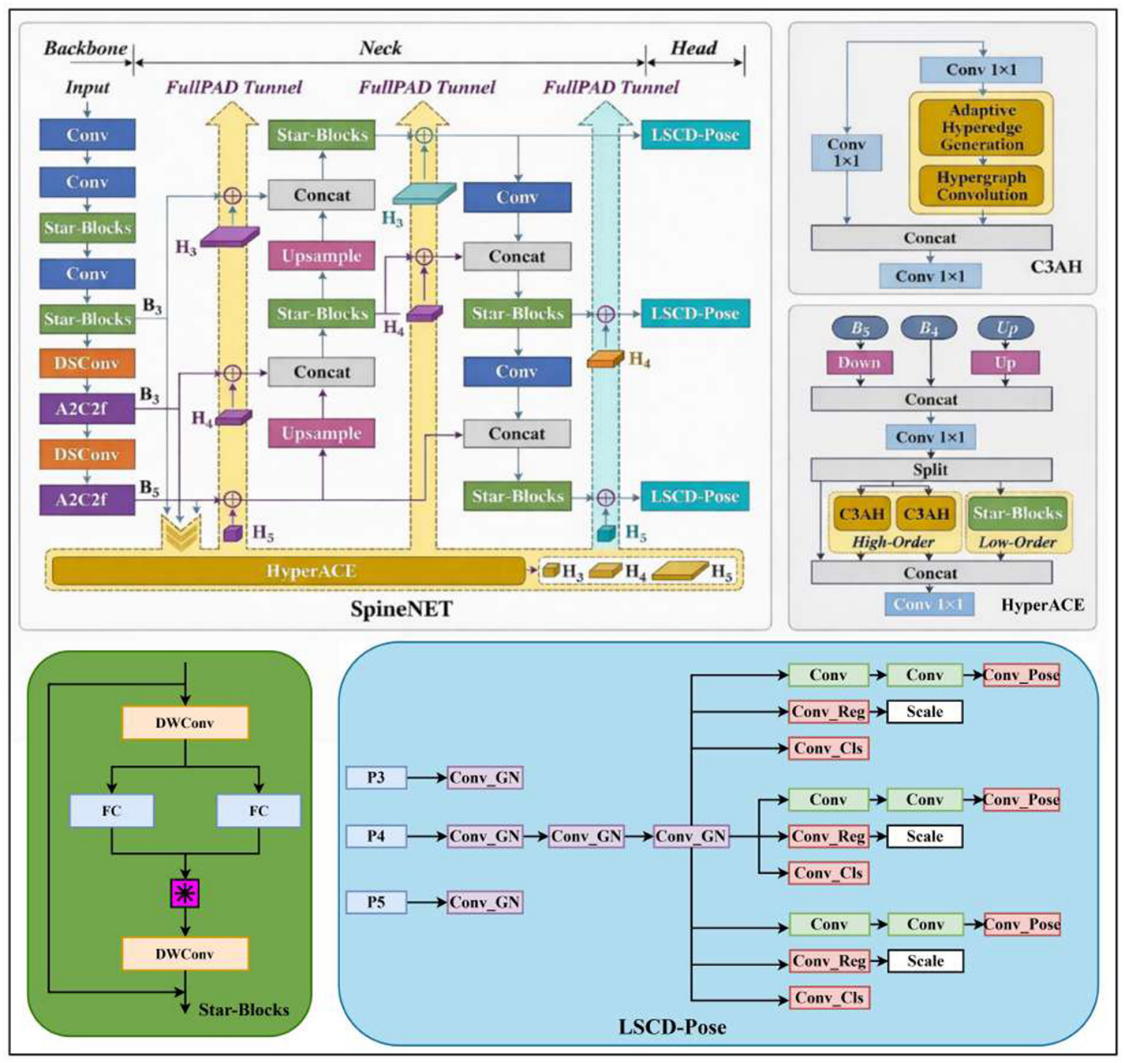
Overview of the proposed SpineNET architecture. This YOLO13-based framework includes a Star-Block-optimized backbone/neck and a lightweight shared-convolution detection head.

SpineNET follows the YOLO13 detection paradigm and leverages its multi-scale feature pyramid with combined top-down and bottom-up fusion pathways to cover vertebral targets across a wide range of scales. An overview of the SpineNET architecture is shown in Figure 2.

Architecturally, SpineNET uniformly replaces the original DS-C3k2 modules in both the backbone and neck with Star-Blocks derived from StarNet (14). The key operation is the Star Operation, implemented as an element-wise multiplication (Hadamard product) between the outputs of two linear transformation branches, which strengthens feature representation and facilitates cross-scale information interaction.

Let the input feature be *X*. The Star Operation can be formulated as Equation 1


$$\begin{array}{l} Y=\left(W_{1}^{⊤}X+b_{1}\right)⊙\left(W_{2}^{⊤}X+b_{2}\right), \end{array}$$ (1)

where ⊙ denotes the Hadamard product. Prior studies have shown that this element-wise multiplicative interaction can enhance nonlinear representation capacity without explicitly increasing channel width, by implicitly modeling feature interactions and expanding the effective feature space.

For spine radiographs, which are characterized by highly repetitive anatomical patterns, blurred boundaries, and low contrast, Star-Blocks can strengthen the extraction of edge- and endplate-related cues with a relatively small parameter overhead, thereby improving the model's sensitivity and stability in perceiving endplate orientation and other geometry-critical attributes.

To further reduce computational complexity and improve the stability of multi-scale predictions, SpineNET adopts a lightweight shared-convolution design (LSCD) detection head. In conventional multi-scale detectors, each pyramid level (e.g., P3, P4, and P5) is typically equipped with an independent convolutional head, which can introduce parameter redundancy and additional computation. LSCD balances accuracy and efficiency through three key designs:

**Shared convolution weights**: detection branches across pyramid levels reuse a single set of convolutional parameters, encouraging the model to learn scale-invariant geometric representations of vertebrae while reducing parameters and FLOPs.

**Adaptive calibration via a scale layer**: a learnable scale factor is introduced for each pyramid level to recalibrate multi-scale responses produced by the shared weights, mitigating amplitude mismatch caused by variations in target size.

**Group normalization (GN)**: GN is used in place of Batch Normalization (BN) to improve training stability and generalization, particularly in medical imaging settings and under cross-center distribution shifts.

### 2.3 From keypoints to clinical measurements

The output layer of SpineNET directly predicts bounding boxes and the corresponding anatomical keypoint coordinates for vertebral and pelvic structures. All clinically relevant quantitative measurements are generated at inference time by a dedicated geometric computation module based on these keypoints, ensuring a transparent, traceable, and clinically auditable measurement process.

For the *n*-th vertebra, the network predicts four corner keypoints *l*_*t, n*_, *r*_*t, n*_, *l*_*b, n*_, *r*_*b, n*_ (subscripts *t* and *b* denote “top” and “bottom” corresponding to superior and inferior endplates, respectively). The superior and inferior endplate orientations are approximated by the line segments *l*_*t, n*_→*r*_*t, n*_ and *l*_*b, n*_→*r*_*b, n*_, with angles (relative to the horizontal axis) computed as Equations 2 and 3:


$$\begin{array}{l} \alpha_{n}=\arctan2\left(r_{t,n,y}-l_{t,n,y},\;r_{t,n,x}-l_{t,n,x}\right), \end{array}$$ (2)

$$\begin{array}{l} \beta_{n}=\arctan2\left(r_{b,n,y}-l_{b,n,y},\;r_{b,n,x}-l_{b,n,x}\right), \end{array}$$ (3)

where *r*_*t, n, x*_/*r*_*t, n, y*_ represent the *x*-/*y*-coordinates of the top-right keypoint of the *n*-th vertebra (consistent indexing for other keypoints). Angles are normalized to [−90, 90], and the vertebral tilt angle is defined as Equation 4:


$$\begin{array}{l} \theta_{n}=\frac{\alpha_{n}+\beta_{n}}{2}. \end{array}$$ (4)

Given a detected vertebral sequence [*s, e*] (where *s* and *e* denote the start and end indices of visible vertebrae), we search for an end-vertebra pair (*i, j*) that maximizes the Cobb angle magnitude Equation 5:


$$\begin{array}{l} C\left(i,j\right)=|\alpha_{i}-\beta_{j}|. \end{array}$$ (5)

The pair (*i, j*) with the largest *C*(*i, j*) is selected as the end vertebrae for the curve segment. A minimum span constraint (e.g., *j*−*i*≥3) is enforced to avoid short-range spurious selections, and the procedure terminates if the maximum Cobb angle is < 5° or if the interval is too short. The same search is then applied to the remaining superior and inferior intervals, yielding up to three Cobb angles for standing whole-spine radiographs and typically one for thoracic- or lumbar-view radiographs.

To achieve accurate vertebral level numbering under truncated field-of-view (FOV) conditions, we established a fixed anatomical alignment rule based on pelvic landmarks: the bilateral PSIS keypoints are used as the anatomical anchor for the inferior boundary of the lumbar spine, and the vertebra immediately superior to the PSIS horizontal line is defined as the 5th lumbar vertebra (L5). The remaining visible vertebrae are numbered sequentially in the superior direction (L4 to T1) based on the L5 anchor. For thoracic-only FOV images without pelvic visibility, we number the visible vertebrae sequentially from the most superior visible vertebra (usually T1) in the inferior direction, and the final level numbering is verified via the sequential distribution of vertebral aspect ratios (thoracic vertebrae have a larger height-to-width ratio than lumbar vertebrae). This alignment rule ensures consistent vertebral numbering across different FOV types and imaging centers.

## 3 Experiments and results

### 3.1 Experimental setup

All experiments were conducted on a high-performance workstation equipped with an Intel Xeon Platinum 8255C CPU and an NVIDIA GeForce RTX 4090 GPU, running Ubuntu 18.04.5 with CUDA 11.1. The models were implemented and trained using PyTorch.

Training hyperparameters were set as follows: 200 epochs, a batch size of 16. All input radiographs were preprocessed with adaptive padding to preserve the original aspect ratio, then resized to a unified input resolution of 640 × 640. Data loading used 8 worker threads, and the initial learning rate was set to 0.01.

### 3.2 Performance evaluation of SpineNET

To comprehensively evaluate SpineNET on the spinal keypoint detection task, we conducted comparative experiments against representative baseline models and performed ablation studies to quantify the contributions of individual components.

#### 3.2.1 Comparison with existing models

Table 2 summarizes the performance of SpineNET and competing methods in terms of keypoint detection accuracy (mAP), model size (parameters), and computational complexity (FLOPs). SpineNET achieved 94.2% mAP@50 and 85.7% mAP@50–95, while maintaining a lightweight footprint (1.7M parameters; 4.3B FLOPs). For instance, YOLO13n reached 89.9% mAP@50 and 80.3% mAP@50–95. Although YOLO13n involves fewer parameters and lower computational cost, SpineNET provides higher detection accuracy and preserves relatively low inference overhead, demonstrating a favorable accuracy–efficiency trade-off.

**Table 2:** Performance comparison of SpineNET and existing models on keypoint detection.

Model	mAP@50	mAP@50–95	Params (M)	FLOPs (B)
ViTPose-s	74.4%	66.7%	40.1	8.2
RTMPose-t	76.3%	68.9%	3.4	4.3
YOLOv5n	75.9%	64.8%	2.1	5.0
YOLOv8n	82.6%	72.6%	3.2	8.7
YOLOv10n	79.3%	68.5%	2.3	6.7
YOLO11n	81.6%	73.3%	2.9	7.4
YOLO12n	84.9%	73.9%	2.8	7.1
YOLO13n	89.9%	80.3%	2.6	6.5
SpineNET (Ours)	94.2%	85.7%	1.7	4.3
YOLO13n+StarBlocks	91.3%	82.8%	1.9	5.1
YOLO13n+LSCD	93.8%	83.6%	2.4	5.7

#### 3.2.2 Ablation study: contributions of Star-Blocks and LSCD

To quantify the contributions of Star-Blocks and the LSCD head, we conducted ablation experiments (Table 3).

**Table 3:** Ablation study on the contributions of Star-Blocks and LSCD.

Model	mAP@50	mAP@50–95	Params (M)	FLOPs (B)
YOLO13n	89.9%	80.3%	2.6	6.5
YOLO13n+StarBlocks	91.3%	82.8%	1.9	5.1
YOLO13n+LSCD	93.8%	83.6%	2.4	5.7
SpineNET (Ours)	94.2%	85.7%	1.7	4.3

Starting from the YOLO13n baseline, replacing the corresponding modules in the backbone and neck with Star-Blocks improved detection accuracy to 91.3% mAP@50 and 82.8% mAP@50–95, while reducing the model size to 1.9M parameters. Alternatively, replacing the original detection head of YOLO13n with LSCD yielded 93.8% mAP@50 and 83.6% mAP@50–95, with 2.4M parameters. When combining Star-Blocks and LSCD (i.e., SpineNET), performance further increased to 94.2% mAP@50 and 85.7% mAP@50–95, while maintaining a lightweight footprint (1.7M parameters; 4.3B FLOPs), indicating a favorable accuracy–efficiency trade-off.

Overall, the ablation results suggest that both Star-Blocks and LSCD contribute to improved keypoint detection performance, and their combination yields additional gains in both accuracy and efficiency.

**Figure 3:**
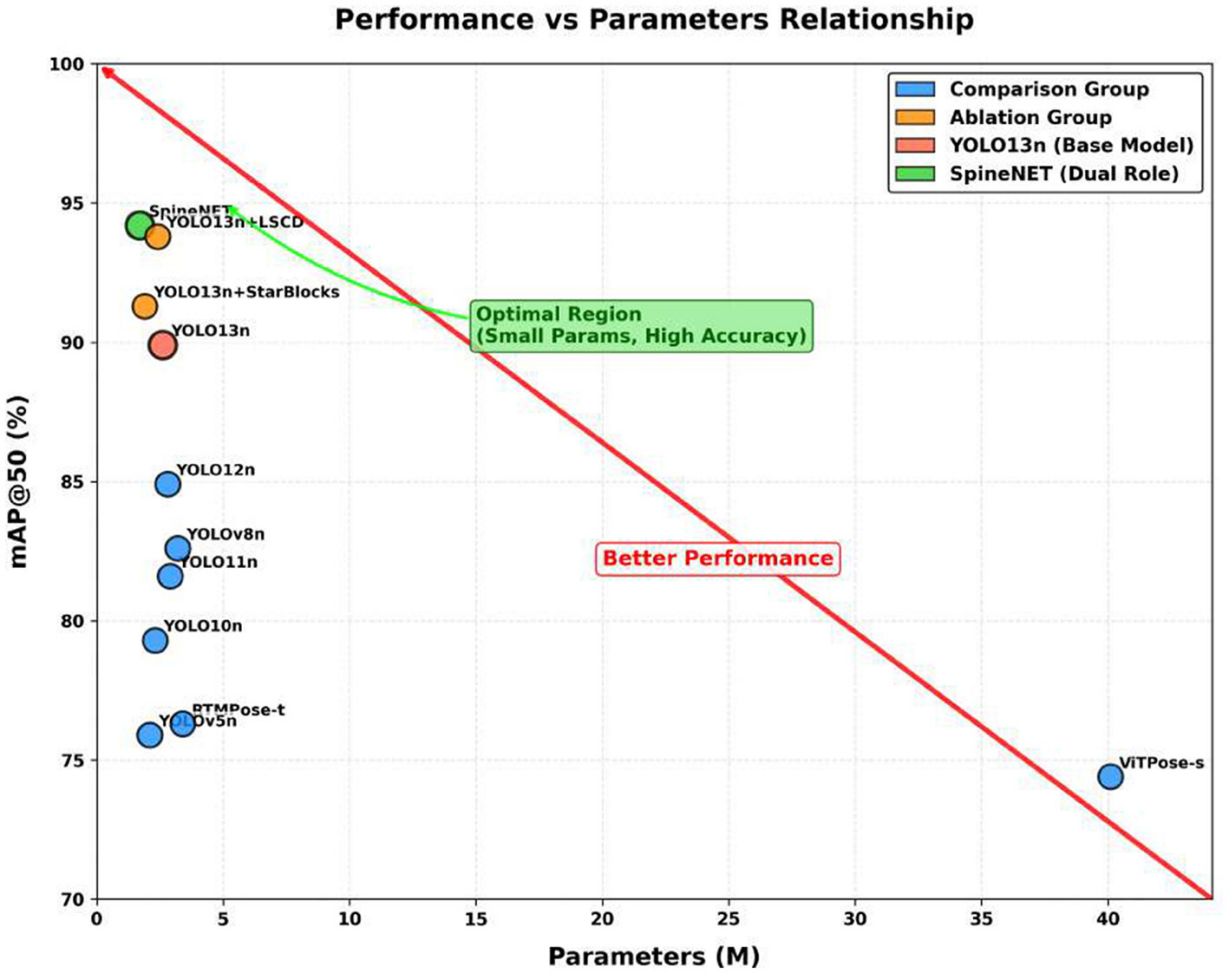
Performance comparison of keypoint detection accuracy (mAP@50) against model parameter count across all evaluated models.

Figure 3 compares SpineNET with baseline methods in terms of keypoint detection accuracy (mAP) and model size (parameters). Overall, SpineNET achieves higher mAP while maintaining a relatively small parameter budget, indicating a favorable accuracy–efficiency trade-off.

### 3.3 Keypoint-based measurement-assisted system for scoliosis assessment

**Figure 4:**
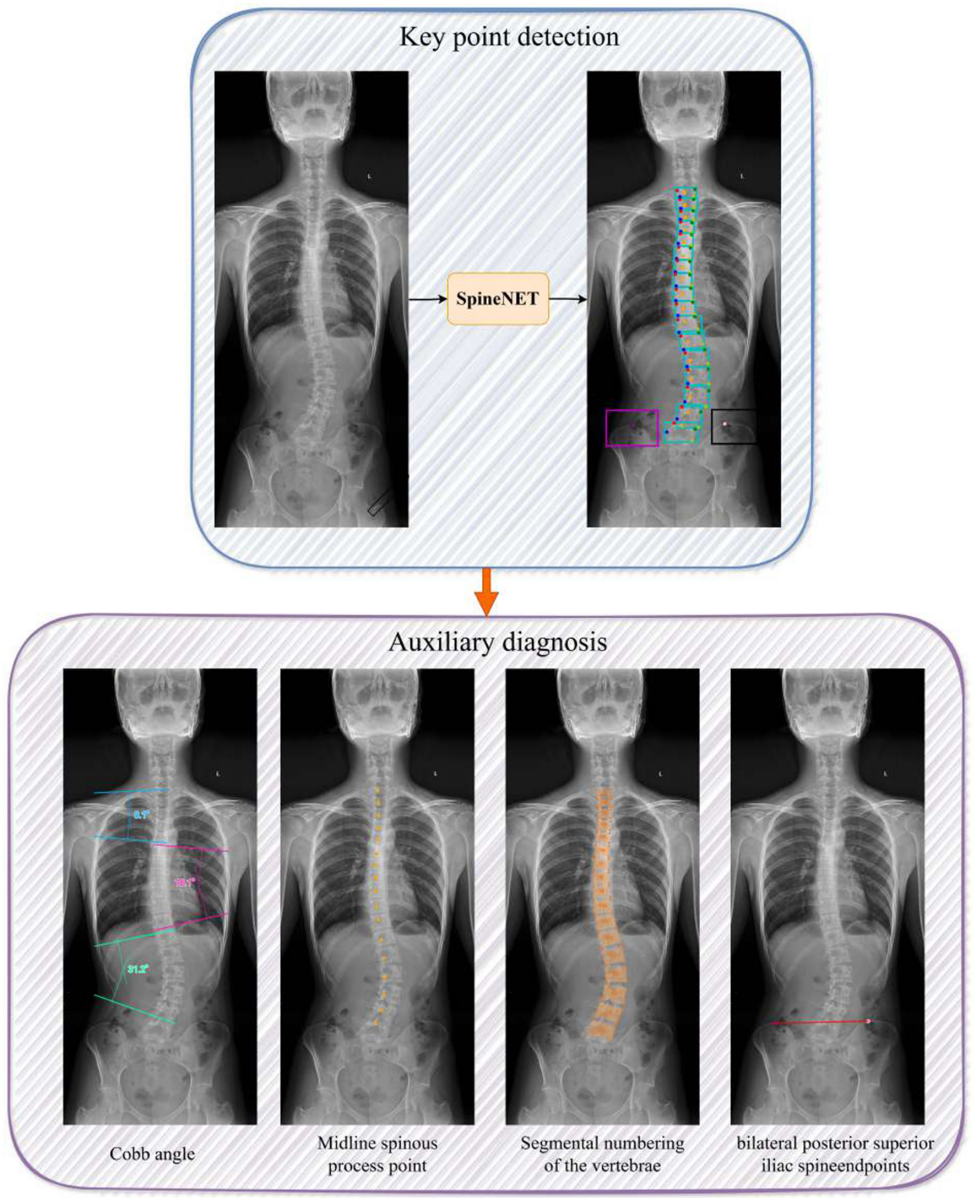
Representative outputs of SpineNET, including keypoint detection visualization and automatically generated auxiliary diagnostic metrics for scoliosis assessment.

In SpineNET, after keypoint detection, a geometric computation module generates multiple clinically auditable, measurement-assisted outputs at inference time, including visual overlays of predicted landmarks and the corresponding quantitative clinical measurements. These outputs facilitate rapid assessment of coronal spinal alignment and related radiographic indicators. Representative examples are shown in Figure 4.

First, based on the predicted keypoint coordinates, the system computes the Cobb angle at inference time via geometric computation, providing a quantitative measure of curve severity. Automated measurement can shorten the time to obtain angle estimates and reduce subjective variability associated with manual readings. Second, SpineNET outputs vertebra-level anatomical landmarks, including the midline spinous-process keypoints, which provide supportive cues for radiographic assessment of scoliosis morphology and rotation-related presentation; the visual overlay of keypoints further enables rapid clinical auditability. In addition, the system assigns vertebral level labels for visible vertebrae, facilitating fast identification of vertebral levels on standing whole-spine radiographs. Finally, by detecting the bilateral PSIS endpoints, SpineNET provides clinically useful information for assessing pelvic coronal tilt.

### 3.4 Comparison between automatically computed Cobb angles and expert measurements

First, we established a standardized manual measurement protocol in accordance with the clinical guidelines of the Scoliosis Research Society (SRS). Three senior spine surgeons (each with more than 10 years of clinical experience in scoliosis diagnosis and treatment) were recruited, and all were blinded to the model's measurement results and other readers' measurements during the independent reading process. Each surgeon independently completed end-vertebra selection and Cobb angle measurement for all 150 external test radiographs using a professional medical image viewing system. After independent measurement, the three surgeons held a joint review meeting for all cases: for cases where the maximum difference between the three independent measurements was ≤ 3°, the arithmetic mean of the three measurements was taken as the final consensus reference value; for cases where the maximum difference exceeded 3°, the three surgeons jointly re-evaluated the radiograph, re-selected the end vertebrae, and reached a unanimous consensus on the final Cobb angle value. The consensus reference values were used as the gold standard for subsequent model performance validation.

**Figure 5:**
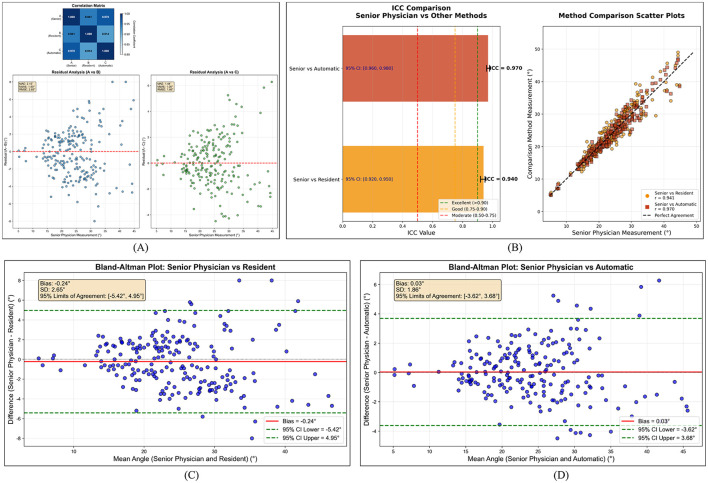
Comparison of SpineNET-derived Cobb angles with the expert reference: error metrics, ICC, and Bland–Altman agreement analysis. **(A)** Residual analysis; **(B)** ICC comparison; **(C)** Bland–Altman plot: senior physician vs. resident; **(D)** Bland–Altman plot: senior physician vs. automatic.

To validate the accuracy of SpineNET for automated Cobb angle measurement, we compared model-derived Cobb angles with a reference established by three senior spine surgeons. The three experts independently measured Cobb angles for each case and reached a final reference value via a consensus procedure. To further assess performance in routine clinical reading scenarios across experience levels, we also compared SpineNET with measurements from a mid-level physician. The results are summarized in Figure 5.

As shown in Figure 5A, SpineNET achieved low measurement errors relative to the expert reference, with a mean absolute error (MAE) of 1.44°, a median absolute error (MdAE) of 1.08°, and a root mean square error (RMSE) of 1.86°. These errors were lower than those between the mid-level physician and the expert reference (MAE: 2.15°, MdAE: 1.80°, RMSE: 2.65°). The correlation heatmap further indicated the highest agreement between SpineNET and the expert reference (correlation coefficient 0.970).

We additionally quantified agreement using the intraclass correlation coefficient (ICC). As shown in Figure 5B, the ICC between SpineNET and the expert reference was 0.970, compared with 0.940 between the mid-level physician and the expert reference. The scatter plots also suggest that SpineNET measurements more closely follow the expert reference across the measured range.

Figure 5C presents Bland–Altman analysis. The mean bias between SpineNET and the expert reference was 0.03°, with an SD of 1.86° and 95% limits of agreement (LoA) of [−3.62°, 3.68°], which were overall narrower than those of the mid-level physician.

We further performed subgroup analysis stratified by AIS severity per clinical diagnostic criteria. Model performance across mild (Cobb angle < 20°), moderate (20°–35°), and severe (>35°) subgroups is summarized in Table 4.

**Table 4:** SpineNET performance across AIS severity subgroups.

Subgroup	MAE (°)	ICC	Mean bias (°)	95% LoA (°)
Mild (< 20°)	1.12	0.968	0.02	[−2.18, 2.22]
Moderate (20°–35°)	1.51	0.972	0.04	[−3.05, 3.13]
Severe (>35°)	2.27	0.951	0.08	[−4.36, 4.52]

Collectively, these results indicate that SpineNET provides high agreement with the expert reference in this cohort across a range of Cobb angles, with relatively concentrated error distributions. Although errors tended to increase in large-angle cases (>35°), the overall dispersion remained limited. These findings support SpineNET as a reliable measurement-assisted tool for radiographic assessment of spinal deformity.

## 4 Discussion

In this study, we developed and validated a measurement-assisted scoliosis assessment system based on a lightweight keypoint detector, SpineNET. The results indicate that SpineNET achieves high keypoint detection accuracy with a small parameter budget and low computational cost, and it demonstrates high agreement with the expert reference in clinical reliability analyses, with overall errors lower than those observed between a mid-level reader and the expert reference in our cohort.

### 4.1 Technical advantages and lightweight design

Prior approaches to automated Cobb angle measurement often trade off efficiency (e.g., direct regression) against interpretability (e.g., segmentation- or geometry-based pipelines). SpineNET adopts a keypoints-to-geometry strategy that produces auditable evidence (end-vertebrae cues and endplate-related orientations) for clinical review. Moreover, the combination of Star-Blocks and the LSCD head strengthens cross-scale representation while preserving lightweightness across feature extraction, multi-scale fusion, and prediction, leading to an improved accuracy–efficiency balance in ablation studies.

### 4.2 Comparison with prior automated Cobb angle methods

Relative to published automated measurement systems, SpineNET shows competitive accuracy and agreement in this cohort. For example, a clinical study based on deep learning keypoint detection reported a mean absolute error of approximately 2.2°±2.0° for landmark positioning relative to expert annotation and very high ICC (approximately 0.994). A recent full-spine framework reported a mean major-curve error of 3.918° with an ICC of 0.943 in a reader study (15). Other studies have also reported high ICC values (e.g., around 0.973) under specific imaging conditions (16). Collectively, these reports suggest strong progress in the field, while highlighting that performance remains sensitive to dataset characteristics, field-of-view truncation, and annotation protocols. SpineNET adds evidence that stable measurement can be achieved under cross-center and resource-constrained settings.

### 4.3 Clinical reliability and human–AI agreement

Clinical utility depends not only on average error but also on measurement reproducibility. Reliability studies have shown that digital Cobb angle measurement still exhibits non-negligible inter-observer variability (e.g., an average of ~3°), and end-vertebra selection can vary across readers. By combining systematic geometric search over vertebral sequences with clinically auditable visual overlays, SpineNET reduces subjectivity in end-vertebra selection. In our study, SpineNET achieved an MAE of 1.44° and an ICC of 0.970 vs. the expert reference, with lower overall discrepancy than the mid-level reader vs. the same reference, supporting its use as a reliable measurement-assisted tool in screening and outpatient workflows.

### 4.4 Deployment value and high-throughput screening potential

With a lightweight footprint (e.g., ~1.7M parameters), SpineNET is well-suited for deployment in resource-limited environments and can be integrated into routine reading workflows. The system provides standardized overlays and quantitative measurements for initial screening, verification, and longitudinal follow-up, improving consistency and efficiency in high-throughput settings. While instance segmentation–based pipelines are also feasible, the keypoint-centric, geometry-driven outputs of SpineNET offer a direct and auditable representation that facilitates quality control and clinical review.

### 4.5 Limitations and future work

This study has several limitations. First, model errors increased in severe AIS cases (Cobb angle >35°). This is mainly driven by severe vertebral deformity, overlapping anatomical structures, and blurred endplate visibility in radiographs of severe curves, which increase the difficulty of stable keypoint localization. Future work will improve robustness in severe cases via targeted data augmentation for severe deformity, incorporation of spinal anatomical structural priors, and enhanced cross-scale feature modeling. Second, this study focused solely on coronal posteroanterior (PA) radiographs, the standard imaging modality for AIS initial screening and routine follow-up in resource-constrained settings. We fully acknowledge the clinical importance of sagittal spinal parameters and Lenke classification for comprehensive AIS evaluation and surgical decision-making in specialized centers. Given the core objective of developing a lightweight, efficient tool for high-throughput AIS primary screening—where coronal Cobb angle measurement is the foundational first-line indicator—Lenke classification data were not annotated in our study cohort, which was curated to align with real-world screening workflows prioritizing coronal curve quantification. Future work will expand the dataset to include standardized Lenke classification and sagittal parameter annotations, and extend the model to biplanar radiographs for comprehensive 3D spinal deformity assessment. Third, our dataset focuses on children and adolescents, and further multi-center validation is needed to assess generalizability to adult degenerative scoliosis with complex degenerative changes.

## 5 Conclusion

This study proposes and validates SpineNET, an efficient deep learning framework for accurate assessment of adolescent scoliosis. SpineNET integrates Star-Blocks to enhance nonlinear feature interactions and adopts a lightweight shared-convolution detection head (LSCD) to achieve high-precision keypoint localization under a low computational budget. In our experiments, SpineNET-derived Cobb angles showed high agreement with the expert reference, achieving an MAE of 1.44° and an ICC of 0.970; in this cohort, the discrepancy between SpineNET and the expert reference was lower than that observed between a mid-level clinician and the same reference.

Beyond automated Cobb angle estimation, SpineNET provides a keypoint-driven, geometry-based set of clinically auditable measurement outputs, offering quantitative and reviewable evidence to support follow-up assessment, rehabilitation planning, and treatment decision-making. Its compact footprint and cross-center external evaluation suggest potential for deployment in high-throughput screening and resource-constrained clinical settings, with the aim of improving early detection efficiency and measurement consistency for adolescent scoliosis.

## Data Availability

The raw data supporting the conclusions of this article will be made available by the authors, without undue reservation.
